# Pain management in the emergency department and its relationship to patient satisfaction

**DOI:** 10.4103/0974-2700.70749

**Published:** 2010

**Authors:** La Vonne A Downey, Leslie S Zun

**Affiliations:** Roosevelt University/Health Services, 430 Michigan Ave., Chicago, IL 60605, USA

**Keywords:** Emergency medicine, pain management, customer service

## Abstract

**Background:**

Pain is the most common reason due to which patients come to the emergency department (ED).

**Aim:**

The purpose of this study was to measure the correlation, if any, between pain reduction and the level of satisfaction in patients who presented to the ED with pain as their chief complaint.

**Materials and Methods:**

This study used a randomly selected group of patients who presented to the ED with pain of 4 or more on the Visual Analogue Pain Scale (VAS) as their chief complaint to a level one adult and pediatric trauma center. Instruments that were used in this study were the VAS, Brief Pain Inventory (BPI), and the Medical Interview Satisfaction Scale (MISS). They were administered to patients by research fellows in the treatment rooms. Statistical analysis included frequencies, descriptive, and linear regression. This study was approved by the Internal Review Board.

**Results:**

A total of 159 patients were enrolled in the study. All patients were given some type of treatment for their pain upon arrival to the ED. A logistic regression showed a significant relationship to reduction in pain by 40% or more and customer service questions.

**Conclusions:**

A reduction in perceived pain levels does directly relate to several indicators of customer service. Patients who experienced pain relief during their stay in the ED had significant increases in distress relief, rapport with their doctor, and intent to comply with given instructions.

## INTRODUCTION

Pain is the most common reason due to which patients come to the emergency department (ED).[1] At least 75% of patients present to the ED with a chief complaint related to pain. Pain is the third most common healthcare problem. It has been shown to be more debilitating than both the top two problems of heart disease and cancer. Management of pain in the ED reportedly has not been done very well.[1–3] The Joint Commission on Accreditation of Hospitals Organization (JCAHO) has mandated that there be an effective assessment and treatment of pain, calling it the fifth vital sign.[3] In response to the assertion that EDs have underused analgesics or oligoanalgesia, JCAHO has revised its standards for pain assessment and management.[4]

Several studies have shown that patients rate their satisfaction as very good with the treatment they received in the ED despite receiving minimal pain relief.[3–4] However, other studies have shown that what patients respond to in relationship to customer surveys is the way in which the ED staff responds to their report of pain, not to the actual reduction in pain levels.[4] In as much as patient satisfaction depends on several variables, pain management is an important one, but not the sole reason for patient satisfaction. The overall environment of the ED, the physicians, and nurses are the key components in patient satisfaction.[4]

The focus on patient satisfaction with the ED encounter has been shown to predict compliance with the treatment.[4] The higher the patients’ satisfaction with their specific encounter, the more likely they are to comply with discharge orders and to return to the ED if pain does not dissipate.[4–6] However, a significant number of patients (between 20 and 50%) were not satisfied with the care they received for pain in the ED.[7–12] The ability to improve patient satisfaction has implications for the effectiveness of the prescribed treatment and for the patient’s health status. This finding is important because patient satisfaction is a predictor of health outcomes once the patient leaves the ED.

Survey tools with a range of variables, including questions about facilities to measure customer service indicators, have been used in studies.[8] Patient satisfaction survey was conducted in the patients, 24 hours or more after the patient had left the ED, as part of these studies. In order to capture the patients’ experience more robustly, other studies focused on patient feedback gathered prior to a patient leaving, and focused on patient related problems.[9–11] These studies found that patient satisfaction is a complex variable and should be measured differently in order to better capture all the components that go into patient satisfaction. Two studies re-examined the impact of customer service using the patient’s responses to a more comprehensive patient doctor encounter survey using the Medical Interview Satisfaction Scale (MISS).[12] The higher the patients’ satisfaction with their specific encounter, the more likely they are to collaborate with their treatment plan, to comply with discharge instructions, and to return to the location of their medical encounter if pain does not dissipate.

The purpose of this study was to measure the correlation, if any, between pain reduction and the level of satisfaction in patients who presented to the ED with pain as their chief complaint.

## MATERIALS AND METHODS

This study was conducted for a 10-month period, enrolling patients who presented to an urban level one trauma center with 60,000 annual visits, with a chief complaint of pain of 4 or more on the visual analogue scale (VAS scale). Using EXCEL, a random number lists was generated each day in order to randomly select patients who were eligible to take part in the study. Those under 18 years of age, unable to give consent and not presenting with a chief complaint of pain of 4 or more were excluded from the study. This study was Internal Review Board (IRB) approved.

Three instruments were used in this study. All these instruments were administered by research fellows in the treatment rooms. For the measurement of initial and 1-hour follow up after treatment was administered, the VAS was given to record pain levels.[12] In addition, patients were given the Brief Pain Inventory (BPI) to measure the history of pain with regard to intensity of pain, interference from pain, prior medication taken for pain, and the ability to perform daily activities. This inventory asks patients to rate pain on a 0–10 scale with 0 = no pain and 10 = pain as bad as one can imagine it. The BPI has been established to be a valid and reliable tool in numerous settings.[2]

The MISS, the third instrument administered after treatment and prior to the patient’s discharge, was used to measure the patient’s satisfaction with the individual consultation within the ED. It is a survey that consists of 21 questions that break down the patient’s satisfaction with the ED visit into four categories: distress relief, communication comfort, rapport, and compliance intent. The use of this survey to measure customer service allows for a more cohesive understanding of the components that go toward a positive patient doctor encounter versus previous studies which have measured customer service based on a series of wide variables such as throughput times, facilities, and all staff input. It has been shown to demonstrate evidence for concurrent validity and for internal consistency [Appendix A on MISS]. The use of survey data statistical analysis meant that descriptive and logistic regression were used to predict the relationship, if any, pain relief has to customer service outcomes. A logistic regression model was used due to its ability to predict the probability of an occurrence using input that combines dichotomous and interval data. In order to achieve a power of 75, a total of 150 subjects had to be enrolled in the study.

## RESULTS

A total of 200 patients were approached, with 159 patients enrolling in the study. The chief reason given by those not consenting to take part in the study was that they were in too much pain to participate. The sample consisted of 57% females and 43% males. The top four locations of pain were 36% abdominal, 23% chest, 16% extremities, and 5% other. The vast majority (82%) reported having pain on a scale of 7 or more when they presented to the ED for treatment. All the patients were given some type of treatment for the pain upon arrival to the ED. These treatments did bring about relief for 60% of the patients, with 40% reporting no relief [Table 1 for demographic information]. There was no significant difference based on demographic variables between those who experienced pain relief and those who did not, during their stay in the ED [Table 2 for breakdown between the two groups]. The impact of pain on most patients interfered with the activities of daily living. The results of the BPI showed this to be especially true for patients who rated their pain at 7–10, which made up 82% of the survey population [Table 3].

**Table 1:** Demographic information

Patient characteristics	%
Level of pain	
4–5	7
5–6	11
7–8	39
9–10	43
Race	
African-American	63
Hispanic	23
Other	14
Age (years)	
18–35	45
36–45	19
46–55	23
>55	13
Gender	
Male	43
Female	57
Treatment of pain	
NSAIDs	48
Opioids	33
Anti-emetics	8
Nitroglycerine	7
GI cocktail	3
Muscle relaxers	1
Pain relief	
Yes	60
No	40

**Table 2:** Comparison of groups by demographic characteristics

Group differences	Pain relief (%)	No pain relief (%)
Age (years)		
18-35	42.6	48.4
>55	12.8	9.4
Primary pain		
Abdominal	40.6	31.3
Chest	24.8	20.3
Extremities	15.8	17.2
Treatment of pain		
NSAIDs	51.5	42.2
Opioids	34.3	31.3

NO SIGNIFICANT DIFFERENCE IS FOUND

**Table 3:** BPI rating of 7–10

Activities of daily living	%
General activity	47.2
Normal work	42.9
Sleep	39.3
Enjoyment	37.4
Mood	35.0
Walking	25.5
Relations with others	11.7

The overall results of the MISS survey were positive with the majority (60%) stating that the doctor was interested in them as a person, seemed warm and friendly (65%), took their problems seriously (68%), and they felt free to talk to the doctor about private matters (55%), and the doctor gave advice that was easy to follow (73%). It did capture a few negatives about the patient’s ED experience in that they did not feel that the physician let them say everything they wanted to about their problems (58%) and did not fully understand them (75%). One limitation, however, is that for 8 of the 21 questions, 15–20% of patients were uncertain of the answers. However, when looking at pain reduction and survey results, the logistical regression model did show a significant relationship between relief of pain by 40% or more and interaction with the physician. The selection of 40% or more pain reduction was used to comply with the guidelines for pain reduction put forth by JCAHO (4). The significant questions were as follows: “After talking to doctor I know how serious my illness is” (*P* = 0.03), “The doctor told me all I wanted to know about my illness” (*P* = 0.01), “The doctor seemed warm and friendly to me” (*P* = 0.00), “This is a doctor I would trust with my life” (*P* = 0.01), “The doctor seems to know what he/she is doing” (*P* = 0.03). Patients who did not experience relief of their pain reported having had a relationship with the doctor “not allowing me to talk about my problem” (*P* = 0.01), “not certain why I came in” (*P* = 0.01), and were “not sure the treatment is worth the trouble” (*P* = 0.01).

## DISCUSSION

This study does not support the conclusions of those who had argued that pain relief and customer satisfaction were not directly correlated within the patient’s satisfaction of their ED encounter.[2–7] The use of the MISS survey tool, which broke down the components of the encounter, allowed for a more precise view of what was occurring. The effectiveness of this customer service survey might have also increased in part because the majority of patients did have relief of 40% or more of their pain symptoms during their encounter.

Taken as a whole, the patients’ responses to the MISS survey were mixed. The majority stated that the doctor was interested in them as a person (60%), seemed warm and friendly (65%), took their problems seriously (68%), they felt free to talk to the doctor about private matters (55%), and the doctor gave advice that was easy to follow (73%). They did not, however, feel that the physician let them say everything they wanted to about their problems (58%) or fully understood them (75%). For 8 of the 21 questions, there were 15–20% of patients who were uncertain of the answers, suggesting that a large portion of patients might not have fully understood either the questions or the information given to them by their physician during their ED encounter. These questions were within each of the domain areas which negatively impacted the ability and usefulness of a score within each domain area.

We observed from this study that a positive impact on customer service questions in the MISS survey was in important areas of distress relief, rapport, and compliance intent. The patients’ knowledge was increased with regard to what was occurring by making them understand the seriousness of their illnesses and providing them everything they wanted to know about those illnesses. It also increased their positive regard for the physicians involved in that the patients felt the physicians to be warmer and friendlier, understanding, and the patients could trust the physicians with their lives, and believed that the physicians knew what they were doing. As a result, the patients stated that they were more likely to follow the advice given. These findings confirm previous studies which showed a relationship between increased customer service and patients’ intent on following medical advice.[4]

As seen in other studies, there were a number of patients (40%) who either did not have relief or experienced less than a 40% reduction in pain during their ED visits.[6–10] This statistics imply that almost two out of five patients who came to the ED do not experience a positive change in what JCHCO refers to as the “fifth vital sign.” The reasons behind this finding are not fully covered within this study. However, we did see that it impacts how those patients felt about their encounter within important areas.

### Limitations

This study was conducted at one site, an inner city ED with a skewed population. A convenience trial of patients is always a limitation. A study comparing customer service, pain, and other interventions would be more useful if it could be done over several diverse sites. Further studies should also examine why a large number of patients did not receive significant pain relief, as this study was not able to determine the reasons for this outcome. This study was unable to perform subset analysis due to low numbers in some categories such as the difference between patients with low and high levels of pain, diversity in treatment and cause of pain. It was also skewed toward patients with severe pain since the majority of patients presented with pain scale rating of 7 or above. All these factors could have biased results toward a specific type of patient presenting in the ED.

## CONCLUSIONS

The MISS survey did give a more comprehensive view of customer service; however, 20% of respondents answering “not sure” for 8 out of 21 questions strongly suggest some confusion or misunderstanding of the questions and limited the ability to use the questions to generate a score for each domain area. A study using another customer service survey and the MISS might be useful to determine if it is the survey tool or other issues such as the educational levels of subjects that impacted the uncertain responses.

Patients who had a reduction in pain during their ED visit did change how they felt about their patient encounter. It appeared to positively impact the perceived level of doctor patient communication about the pain, increase the patient rapport with the physician and whether or not the patient would comply with the medical advice given during the encounter [Table 4].

**Table 4:** Comparison between those with and without pain relief

		d.f.	Mean square	F	Significance
MD told me what trouble is	Between groups	1	0.704	0.394	0.531
I know just how serious my illness is	Between groups	1	1.589	1.009	0.317
MD told me all I wanted to know	Between groups	1	0.891	0.471	0.493
Not certain how to follow MD advice	Between groups	1	3.461	2.351	0.127
Good idea how long before well again	Between groups	1	0.100	0.072	0.789
MD interested in me as person	Between groups	1	1.691	1.278	0.260
MD warm and friendly to me	Between groups	1	3.810	4.058	0.046
MD take problems seriously	Between groups	1	4.865	3.898	0.050
Felt embarrassed talking with MD	Between groups	1	0.559	0.304	0.582
Free to talk about private matters	Between groups	1	2.024	1445	0.231
MD gave me change to say on my mind	Between groups	1	10.386	5.462	0.021
I felt understood by MD	Between groups	1	0.001	0.001	0.979
MD not allow me to say all about my problems	Between groups	1	2.679	1.944	0.165
MD not understand my main reason for coming	Between groups	1	0.478	0.428	0.514
I would trust MD with my life	Between groups	1	3.116	1.892	0.171
MD really know what is doing	Between groups	1	6.140	4753	0.031
Md relieved my worries about illness	Between groups	1	2.006	2.365	0.126
MD knows what to do for my problem	Between groups	1	1.359	0.932	0.336
Advice easy to follow	Between groups	1	1.173	1.193	0.276
Difficult to do what MD told me	Between groups	1	2.006	1.446	0.231
Not sure MD treatment worth the trouble	Between groups	1	1.613	1.785	0.184

The use of the MISS survey gave a more direct understanding of what areas of customer service the pain reduction impacted. It also showed that there are other areas that are not impacted by a positive physical outcome, such as communication comfort levels between physicians and patients. This gives an indication, as other studies have shown, that simply solving or addressing the patients’ presenting condition will not uniformly increase their customer service indicators. This was seen in this study in those patients who did not experience pain relief during their encounter. Their response to the survey with the exception of the noted questions was similar to patients who did get pain relief. The fact that such a large percentage had little to no pain relief is an indicator that more needs to be done to address the JHACO concerns apart from its impact on customer service indicators.

## Appendix A

**Table:** Medical interview satisfaction scale-21

The patient was asked to indicate their level of agreement on a 7-point Likert scale
Very strongly disagree = 1, strongly disagree = 2, disagree = 3, uncertain = 4, agree = 5, strongly agree = 6, very strongly agree = 7
The doctor told me just what my trouble is (DR) After talking with the doctor, I know just how serious my illness is (DR) The doctor told me all I wanted to know about my illness (DR) I am not really certain about how to follow the doctor’s advice (CC) After talking with the doctor, I have a good idea of how long it will be before I am well again (DR) The doctor seemed interested in me as a person (R) The doctor seemed warm and friendly to me (R) The doctor seemed to take my problems seriously (R) I felt embarrassed while talking with the doctor (CC) I felt free to talk to this doctor about private matters (R) The doctor gave me a chance to say what was really on my mind (R) I really felt understood by my doctor (R) The doctor did not allow me to say everything I had wanted about my problems (CC) The doctor did not really understand my main reason for coming (CC) This is a doctor I would trust with my life (R) The doctor seemed to know what (s)he was doing (R) The doctor has relieved my worries about my illness (DR) The doctor seemed to know just what to do for my problem (DR) I expect that it will be easy for me to follow the doctor’s advice (CI) It may be difficult for me to do exactly what the doctor told me to do (CI) I’m not sure the doctor’s treatment will be worth the trouble it will take (CI)

DR = DISTRESS RELIEF SUBSCALE; CC = COMMUNICATION COMFORT SUBSCALE; R = RAPPORT SUBSCALE; CI = COMPLIANCE INTENT SUBSCALE

## References

[1] Tanabe P, Buschmann M (1999). A prospective study of ED pain management practices and the patients prospective. J Emerg Nurs.

[2] Garbez RO, Chan GK, Neighbor M, Puntillo K (2006). Pain after discharge: A pilot study of factors associated with pain management and functional status. J Emerg Nurs.

[3] Dillard J, Knapp S (2005). Complementary and Alternative Pain Therapy in the Emergency Department. Emerg Med Clin North Am.

[4] Todd KH, Ducharme J, Choiniere M, Crandall CS, Fosnocht DE, Homel P (2007). Pain in the emergency department: results of the pain and emergency medicine initiative (PEMI) multicenter study. J Pain.

[5] Muntlin A, Gunningberg L, Carlsson M (2006). Patient’s perception of quality of care at an emergency department and identification of areas for quality improvement. J Clin Nurs.

[6] Baker R (1990). Development of a questionnaire to assess patients’ satisfaction with consultation. Br J Gen Pract.

[7] Ford R, Bach S, Fottler M (1997). Methods of Measuring Patient Satisfaction in Health Care Organization. Hlth Care Manag Review.

[8] Dougherty C (2005). Customer Service in the Emergency Department. Topics in Emergency Medicine. Adv Emerg Nurs.

[9] Garman A, Garcia J, Hargreaves M (2004). Qual Health Care.

[10] Guru V, Dubinsky I (2000). The Patient VS. Caregiver Perception of Acute Pain in the Emergency Department. J Emerg Med.

[11] Matheson LJ, Melhorn M, Mayer T (2006). Reliability of a Visual Analog Version of the QuickDASH. J Bone and Joint Surg.

[12] Meakin R, Weinman J (2002). The Medical Interview Satisfaction Scale. Fam Prac J.

